# Drug-related problems and their predictors in pediatric community-acquired infections: the role of pharmacist-led interventions in Pakistan

**DOI:** 10.3389/jpps.2026.16612

**Published:** 2026-07-16

**Authors:** Sana Ali, Ale Zehra, Fakhsheena Anjum, Syed Shaukat Ali Muttaqi Shah, Falak Abro, Sadia Shakeel, Muhammad Ali

**Affiliations:** 1 Department of Pharmacy Practice, Faculty of Pharmaceutical Sciences, Dow College of Pharmacy, Dow University of Health Sciences, Karachi, Sindh, Pakistan; 2 Department of Clinical Pharmacy, College of Pharmacy, Al-Farahidi University, Baghdad, Iraq; 3 Department of Pediatrics, Liaquat National Hospital, Karachi, Sindh, Pakistan; 4 Department of Pharmaceutics and Pharmacy Practice, Faculty of Pharmacy, Salim Habib University, Karachi, Pakistan

**Keywords:** community-acquired infections, drug-related problems, economic impact, medication safety, Pakistan

## Abstract

**Purpose:**

Drug-Related Problems (DRPs) are a leading cause of preventable harm to hospitalized children, yet the data from low- and middle-income countries (LMICs) are limited. This study aimed to determine the prevalence, severity and determinants of DRPs in children with community-acquired infections (CAIs) using standard classification, and to quantify clinical and economic impact of pharmacist-led interventions.

**Methods:**

A prospective interventional study conducted (June 2024-March 2025), in pediatric ward of tertiary care public hospital, Karachi, Pakistan. Children aged 2months-14 years with CAIs were enrolled. A trained pharmacist reviewed medication orders daily, identifying DRPs (classified by PCNE V9.1 and severity by NCC MERP criteria). Interventions were proposed to physicians and acceptance rates recorded. Direct cost savings were calculated and DRP predictors were identified using logistic regression.

**Results:**

A total of 3,842 medication orders were reviewed for 400 patients, identifying 2,010 DRPs (5.03 DRPs/patient). Primary causes were dose-selection (C3-58.13%), drug-form (C2-15.01%), and drug-selection (C1-13.08%). Severity assessment classified 9.0% of errors as having potential to cause serious harm. Pharmacist interventions had 98.7% acceptance and 81.1% DRPs were resolved. Number of medications (AOR 1.32), fever (AOR 2.84) and length of stay >7days (AOR 1.76) were identified as predictors of DRPs; past immunization was protective (AOR 0.51). Direct cost saving was PKR 363,184 (USD1,290) over 10 months (23:1 cost ratio). Cost per DRP prevented was PKR 241 (USD 0.86), with 142% return on investment.

**Conclusion:**

DRPs affected 52.3 per 100 medication orders in hospitalized children with CAIs, with dose selection DRPs predominant and 9% having serious harm potential. Pharmacist-led interventions demonstrated a 98.7% acceptance rate and 81.1% resolution of DRPs, and were associated with substantial cost savings, providing evidence for integrating clinical pharmacy services in LMICs.

## Introduction

Drug-related problem (DRP) is defined as “an event or circumstance involving drug therapy that actually or potentially interferes with the desired health outcome in a patient” [1–3]. DRPs encompass a broad range of issues, including prescribing errors, monitoring errors, adverse drug reactions, and patient non-adherence [1–3]. Prescription errors, a subset of DRPs, are defined as a failure in the prescription process that includes incorrect drugs selection, dosage form, frequency, route or duration, and are the leading cause of preventable harm in the hospitalized children [4]. These prescription related DRPs are particularly more dangerous in children as pediatric patients have weight based dosing calculation, age-dependent distribution and excretion of drugs and have a frequent need for the off-label drug use [5]. The prevalence of DRPs ranges from 5 to 68% in pediatric wards, with dose-related DRPs being the most frequently reported type [6, 7].

Community-acquired infections (CAIs) are a leading cause of deaths and hospitalization in the children globally, and the burden of CAIs is particularly high in low- and middle-income countries (LMICs) where infectious diseases are the leading burden of diseases [1, 2]. CAIs account for approximately 60–70% of pediatric hospital admissions in tertiary care centers in Pakistan [8, 9]. Enteric fever, pneumonia and gastroenteritis account for a large proportion of pediatric hospital admissions in Pakistan [8, 9]. Children with CAIs are vulnerable to DRPs due to the frequent use of multiple antibiotics, weight-based dosing calculations, narrow therapeutic indices of antimicrobials, and the need for dose adjustments in febrile and dehydrated states [10, 11]. This makes the group ideal for pharmacist interventions, which could have a significant clinical and economic impact.

The burden of DRPs is higher in LMICs due to factors including: limited healthcare resources; rudimentary clinical pharmacy services; absence of electronic medical records (EMR) and computerized prescriber order entry (CPOE) systems; inconsistent access to pediatric dosing references; and lack of local protocols and guidelines [12, 13]. Countries like Pakistan face even higher risks of DRPs due to lack of human resources (high patient to physician ratios), limited formulary availability and lack of availability of pediatric-specific formulations [14]. In addition to the clinical consequences, DRPs pose a huge economic burden. DRPs are estimated to cost 42 billion dollars annually to healthcare systems, and length of stay is prolonged by 4–6 days due to preventable adverse drug events, adding an additional cost of $2500-$9,000 per event [15, 16]. A systematic review of pediatric antimicrobial stewardship (AMS) programs showed that only 14.1% of studies evaluated the cost outcomes, which highlights a critical evidence gap [17]. Pharmacists practicing in hospital and clinical settings contribute significantly to medication safety through prospective medication order review, identification of DRPs and providing feedback and interventions to the prescribers [18, 19]. Participation of pharmacists in ward rounds reduces DRPs by 50%–80%, resulting in significant cost savings and improved clinical outcome [18, 20, 21].

The Pharmaceutical Care Network Europe (PCNE) has developed a validated classification system (V9.1) that enables systematic identification, categorization, and comparison of DRPs across different settings [22]. Although there is wide use of PCNE classification to identify and classify the DRPs in adult population and some pediatric settings, its use in LMICs remains limited [23]. No prospective study from South Asia has applied PCNE V9.1 classification to quantify DRPs in hospitalized children along with the economic impact of pharmacist interventions using cost data. This gap of classification of DRPs along with rigorous economic analysis represent a critical missing evidence base for clinical pharmacy services in LMICs [24].

The study objectives were to: determine the prevalence, types and causes of DRPs in hospital pediatric patients with CAIs in Pakistan using PCNE V9.1 classification; to assess the severity of DRPs using National Coordinating Council for Medication Error Reporting and Prevention (NCC MERP) criteria; to identify the patient and system level predictors of DRPs; and to evaluate the clinical and economic impact of pharmacist led interventions, based on the actual drug acquisition cost and return on investment.

The study tested three hypotheses: (1) DRPs would affect >40% of medication orders, with dose related DRPs as the major contributor; (2) pharmacist led interventions would achieve ≥90% acceptance; and (3) net cost savings from DRP correction would exceed the cost of pharmacist time, showing positive return on the investment. These data are important to highlight the clinical pharmacy services in LMICs where resource constraints exists and all the finances must be justified. By demonstrating both DRP reduction and cost savings, this study may provide evidence-base data to inform hospital administrators and policymakers for strategic investment in clinical pharmacy services and improving patient safety.

## Materials and methods

### Study design and setting

This prospective interventional study was conducted over a 10-month period from June 2024 to March 2025. A trained pharmacist was stationed within the Pediatric Medical Ward (PMW) and Pediatric High-Dependency Unit (PHDU) of a tertiary care public hospital in Karachi, Pakistan, during the morning shift (0900–1500). Ethical approval for this study was obtained from the Institutional Review Board of Dow University of Health Sciences (Ref: IRB-3464/DUHS/Approval/2024/131). This study was conducted in accordance with the Declaration of Helsinki, a written informed consent in both English and Urdu (local language) was obtained from the parents or legal guardians. Additionally written assent was obtained from children age 7 years and above.

### Participants and eligibility criteria

Children between 2 months and 14 years who were admitted to PMW or PHDU with a confirmed diagnosis of one or more CAIs were eligible for inclusion in the study. The CAIs diagnosis included enteric fever, acute gastroenteritis, pneumonia, dengue fever, malaria, meningitis, urinary tract infections, viral hepatitis, bronchitis, and skin and soft tissue infections. Patients admitted to the Intensive Care Unit (ICU) or Neonatal Intensive Care Unit (NICU) were excluded from the study. Patients with hospital stay less than 24 h (early discharge) were also excluded to ensure that patients received at least one full day of pharmacotherapy, allowing adequate time for medication order review and DRP identification. The patients with incomplete medical records, or those whose medical records were not available due to any reason were also excluded from the study.

OpenEpi (version 3.01) was used to calculate the sample size for a single population proportion. Assuming 50% anticipated frequency of DRPs, with a 5% margin of error and 95% confidence interval, the minimum statistically significant sample requirement was 382 patients [25]. To account for the potential attrition (incomplete records, early discharges), we enrolled 400 patients, providing 80% power to detect a 10% difference in error rates between subgroups with α = 0.05.

### Data collection tool

Data collection utilized a structured form (Supplementary Material 1), which was developed based on previous studies of pediatric DRPs [26, 27]. Data were extracted from electronic medical record and patient files. The form included demographic information (Age, gender, weight, and residential area), clinical characteristics (presenting complaints of the patient, diagnosis, comorbidities, vaccination status, allergy history), laboratory data (Total leukocyte count, Serum Creatinine, liver profile, microbiology culture results etc.), medication profile of the patient (all prescribed drugs, name, dose, frequency, route, duration and indication), and hospitalization data (Length of stay and ward type etc).

### PCNE classification of DRPs

DRPs were classified using the PCNE classification V9.1 [22]. The PCNE framework comprises five domains: Problem (P), which describes the nature of the DRP (e.g., treatment effectiveness, adverse events); Causes (C), which identifies the underlying reason for the DRP (e.g., dose selection, drug selection); Interventions (I), which documents the actions taken to address the DRP; Acceptance (A), which records the prescriber’s response to the intervention; and Outcome (O), which indicates the resolution status of the problem [3]. This classification enables systematic analysis and comparison of data internationally. All identified DRPs were mapped to PCNE codes based on their characteristics, and multiple causes could be assigned to a single DRP where applicable. The PCNE classification used in the study is presented in Supplementary Table 1.

The PCNE V9.1 framework, not only classify errors related to prescription, additionally captures non-prescribing issues such as monitoring errors and patient non-adherence. These DRPs were identified by comparing each medication order against the reference standards: Lexicomp Online® pediatric dosing database, Sanford Guide to Antimicrobial Therapy, WHO treatment guidelines, British National Formulary for Children (BNFc), Hospital formulary and local guidelines. Weight-based dosing guidelines from Lexicomp Online® and BNFc were consistently applied as reference standards for all pediatric medication orders to identify dosing related DRPs.

### Severity classification

The severity of error identified was assessed using the National Coordinating Council for Medication Error Reporting and Prevention (NCC MERP) criteria [28]. The errors were classified as: Minor when an error is unlikely to cause serious harm, permanent damage, or death to the patient (e.g., minor timing issues, incomplete documentation); Moderate when an error is with a potential to require increased monitoring of the patient, or an intervention or prolonged the stay of the patient; and the severe error was an error with potential to cause serious harm, permanent damage or death to the patient.

The PCNE classification was used to identify and categorize the type and cause of DRPs, while the NCC MERP criteria were applied to assess the clinical severity (potential harm) of each identified problem. These two classification systems serve complementary roles: PCNE describes the nature and source of the problem, while NCC MERP quantifies its potential impact on patient safety.

### Blinding and reliability

The primary investigator was not blinded during data collection, presenting a potential source of bias. Non-blinding may have led to overestimation of DRP prevalence, as the primary investigator might have been more likely to identify DRPs knowing the study objectives. To mitigate potential bias from non-blinded assessment, all identified DRPs were verified against reference standards before classification and complex cases were discussed with an Infectious Diseases (ID) specialist for consensus. A 10% random sample of identified DRPs was independently classified by a second pharmacist using PCNE V9.1. Inter-rater reliability was assessed using Cohen’s kappa coefficient: PCNE V9.1 classification yielded κ = 0.85 (95% CI 0.78–0.92), indicating almost perfect agreement, while NCC MERP severity classification yielded κ = 0.82 (95% CI: 0.74–0.90), indicating substantial agreement [29].

### DRP rate calculation

The DRP rates were calculated as; DRPs per 100 prescriptions = (total DRPs/total prescriptions) × 100; DRPs per patient = total DRPs/number of patients; DRPs per 100 patient days = (total DRPs/total length of stay in days) × 100.

### Intervention protocol

The pharmacist (principal investigator) was a regular hospital staff member already working in the pediatric ward. Ward rounds were already part of routine clinical practice before the study period. During this study, the pharmacist continued these routine ward rounds and prospectively collected data for the study. When a DRP was identified, the pharmacist carried out the following interventions: (1) verified the DRP against available reference standards; (2) formulated a specific recommendation based on evidence-based guidelines; (3) communicated the recommendation to the prescribing physician; (4) discussed complex antimicrobial cases with an ID specialist; and (5) documented the intervention and outcome using PCNE codes. This sequential process followed a standardized framework reproducible in other hospital settings.

**FIGURE 1:**
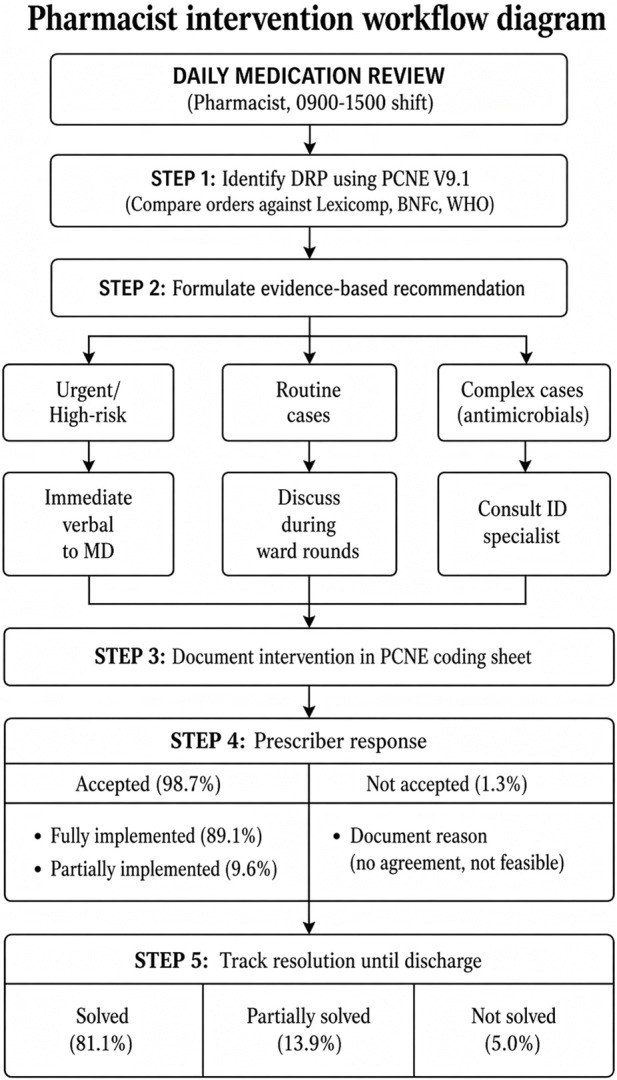
Pharmacist-led intervention workflow diagram. The figure outlines the sequential process from daily medication review to DRP resolution in the study. Steps include: (1) daily medication review and DRP identification using PCNE V9.1 classification; (2) formulation of evidence-based recommendations; (3) communication of recommendations based on urgency; (4) documentation in PCNE coding sheet; (5) prescriber response (accepted: 98.7%; not accepted: 1.3%); and (6) tracking of resolution status (solved: 81.1%, partially solved: 13.9%, not solved: 5.0%).

Communication methods varied based on prescriber availability and urgency. The primary method was verbal discussion during daily ward rounds (0900-1500 shift), where the pharmacist and prescriber reviewed the medication order together (80.4% of prescriber-level interventions). For complex antimicrobial cases, the pharmacist first discussed the recommendation with an ID specialist, who then provided input to the primary team. When the prescriber was not available during rounds, interventions were communicated by phone to the prescriber (10.0% of prescriber-level interventions). All communication methods were documented in the PCNE coding sheet. Intervention acceptance and implementation were distinguished using PCNE V9.1 definitions: ‘accepted’ indicated that the prescriber agreed with the pharmacist’s recommendation in principle, while ‘implemented’ indicated that the recommended change was actually applied to the patient’s medication order. An intervention could be accepted but not implemented due to practical barriers (e.g., patient discharged, or clinical decision to defer change). The status of all interventions was tracked by the pharmacists and coded for: acceptance (A1-accepted, A2 – not accepted); implementation (A1.1 – accepted and fully implemented, A1.2 – accepted but partially implemented, A1.3 – accepted but not implemented, A1.4 – accepted but implementation unknown); and DRP resolution (O1 - solved, O2 - partially solved, O3 - not solved). The standardized intervention workflow is illustrated in Figure 1.

### Cost impact analysis

Direct cost savings were calculated for the interventions that were associated with the reduction of the medication expenses, following the pharmacoeconomic principles [30, 31]. The cost analysis comprised of categories including: wrong dose calculation including dose increase or decrease (C3); duplication of therapy (C1.4); drug formulation changes (C2.1) including IV to oral switch or formulation changes; inappropriate drug choice (C1.1); inappropriate drug combinations (C1.3) i.e., saving from discontinuation of unnecessary medications; dose frequency adjustments (C3.3, C3.4); treatment duration adjustments (C4) including shortened or extended duration of the medication. The detailed formula and categorization of cost impact analysis is presented in Supplementary Material 2. The formula used for cost calculations were; cost savings = recommended drug cost - original drug cost, while drug cost = drug acquisition cost x daily dose x duration of therapy. The cost of the drugs was taken from the standard price list of Drug Regulatory Authority of Pakistan (DRAP) 2023 price list, and the hospital computerized pharmacy records were used to verify acquisition costs. The hospital drug prices remained fixed throughout the study period and all the calculations were based on actual acquisition costs not estimates. All costs are reported in Pakistani Rupees (PKR), with 1 USD ≈280 PKR which is approximate and reflects the exchange rate at the time of study. Purchasing power parity adjustments were not applied, which may affect international comparisons. Cost per DRP prevented was calculated by dividing net cost savings by the total number of corrected DRPs.

This analysis captured only the direct medication cost savings and direct pharmacist intervention costs. Indirect cost savings associated with reduction in length of stay, prevention of adverse drug events, avoiding readmission, decreased nursing time due to dosage form change, and long-term benefits associated with antimicrobial resistance were not quantified. The reported savings serve as estimates of the true impact of pharmacist intervention.

### Return on investment calculation

The return on investment calculations were performed using these formulas: total pharmacist time spent on interventions: estimated 2 h/day × 300 working days = 600 h. The estimate of 2 h per day was derived from daily activity logs maintained by the pharmacist during the study period, which recorded time spent on medication order review, intervention communication, and documentation. This represents the average active intervention time per working day and excludes routine clinical duties unrelated to DRP identification; pharmacist hourly cost (junior hospital pharmacist salary PKR 40,000/month ÷ 160 h) = PKR 250/hour; total intervention cost = 600 h × PKR 250 = PKR 150,000; net saving after deduction intervention cost = cost saving–total intervention cost; return on investment = (net saving after intervention ÷ total intervention cost) x 100. Sensitivity analysis was performed to test the robustness of the economic findings. One-way sensitivity analyses varied the following parameters: (1) pharmacist hourly cost (PKR 200 and PKR 300 instead of baseline PKR 250), (2) pharmacist time spent on interventions (1.5 h and 2.5 h per day instead of baseline 2 h), and (3) drug acquisition costs (±10% variation). Results are reported as range of total cost savings, return on investment, and cost per DRP prevented under these alternative scenarios.

### Outcome measures

The primary outcome was the prevalence of DRPs, measured as DRPs per 100 prescriptions and the percentage of patients with at least one DRP. Secondary outcomes included the acceptance rate of pharmacist interventions (PCNE A domain), DRP resolution rate (PCNE O domain), independent predictors of DRPs (AOR with 95% CI), direct cost savings (total PKR, net savings, cost per DRP prevented), and return on investment ratio. Descriptive classifications (not used as independent outcomes) included the distribution of DRPs by PCNE problem (P) and cause (C) domains, and severity distribution by NCC MERP criteria (minor, moderate, severe).

### Statistical analysis

To ensure data quality and minimize bias, data were double entered by two independent researchers into an Excel spreadsheet. The entries were cross-verified and discrepancies were resolved by rechecking medical records and data sheets. Missing data were handled by complete case analysis, as the proportion of missing data was less than 5% for all variables. Patients with incomplete medical records or missing key variables were excluded from the respective analysis. The verified data were entered into IBM-SPSS version 23.0. Descriptive statistics (frequencies, percentages, means ± standard deviation) were calculated for types of DRPs, PCNE classifications, severity of medication error, interventions, and cost savings. Univariate logistic regression was performed for each predictor variable to calculate crude odds ratios (OR) with 95% confidence intervals. Variables showing significance at p < 0.10 in univariate analysis were considered for entry into multivariate model. The primary outcome for logistic regression was the presence of at least one DRP (binary: 0 = no DRP, 1 = ≥1 DRP). Number of DRPs per patient was used for descriptive purposes only and not as the dependent variable in regression models. Multivariable logistic regression using backward stepwise selection was then performed to identify independent predictors of DRPs, adjusting for potential confounders. The final model included variables with p < 0.05 after stepwise elimination. The events-per-variable (EPV) criterion was satisfied with 372 events (patients with DRPs) and 7 predictor variables, yielding an EPV of 53, which exceeds the recommended minimum of 10. The adjusted odds ratios (AOR) and 95% confidence intervals were reported. A p-value of <0.05 was considered statistically significant for the results.

## Results

### Patient characteristics

A total of 793 pediatric patients were assessed for eligibility, of whom 400 patients were enrolled in the study; the mean was 4.79 ± 4.18 years (range: 0.1–14.0 years) and the mean weight of 16.0 ± 10.4 kg (range: 4.0–55.0 kg). Males comprised 64.5% (n = 258) of the cohort while females were 35.5% (n = 142). A total of 29.5% (n = 118) had a diagnosis of enteric fever, followed by 21.3% (n = 85) with gastroenteritis, 12% (n = 48) with pneumonia, and 10.5% (n = 42) with dengue fever. Most patients 73.5% (n = 294) were receiving more than five medications, 25.5% (n = 102) had 4 to 5 medications prescribed. A total of 65.5% (n = 262) spent an average of 7–9 days in the hospital, while 96% (n = 384) patients were febrile at the time of admission. Table 1 presents baseline characteristics.

**TABLE 1:** Baseline characteristics of study participants (n = 400).

Characteristic	Category	N	%
Gender	Male	258	64.5
	Female	142	35.5
Age (years)	Mean ± SD (range)	4.79 ± 4.18	0.1–14.0
Weight (kg)	Mean ± SD (range)	16.0 ± 10.4	4.0–55.0
Diagnosis	Enteric fever	118	29.5
	Acute gastroenteritis	85	21.3
	Pneumonia	48	12
	Dengue fever	42	10.5
	Malaria	36	9
	Meningitis	32	8
	Urinary tract infections	20	5
	Viral hepatitis	10	2.5
	Bronchitis	9	2.3
Polypharmacy	Minor (2-3 medications)	4	1
	Moderate (4-5 medications)	102	25.5
	Major (>5 medications)	294	73.5
Length of stay	≤6 days	100	25
	7–9 days	262	65.5
	≥10 days	38	9.5
Past immunization	Yes	218	54.5
	No	182	45.5
Fever at admission	Yes	384	96
	No	16	4

Data presented as n (%) unless otherwise specified. SD, standard deviation. Past immunization was defined as receipt of all age-appropriate vaccinations per EPI schedule.

### Prescription volume and DRP rates

**FIGURE 2:**
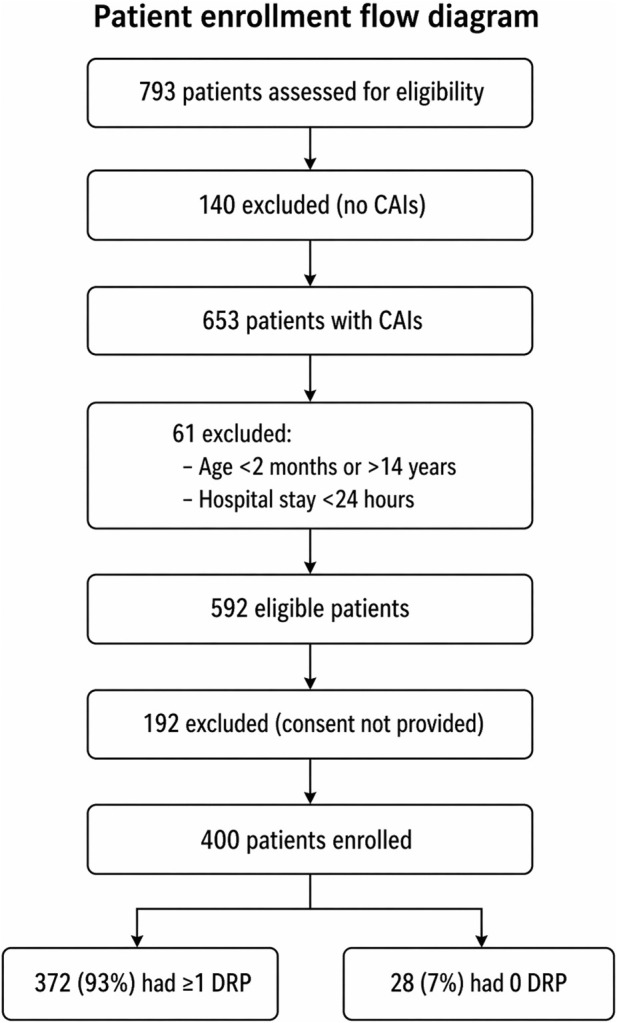
Patient enrollment flow diagram. The flow diagram shows the screening and enrollment process: 793 patients assessed → 140 excluded → 653 with CAIs → 61 excluded → 592 eligible → 192 refused consent → 400 enrolled → 372 (93%) with DRPs and 28 (7%) with no DRPs.

A total of 3,842 medication orders were reviewed across 400 patients (mean of 9.6 orders per patient). A total of 2,010 DRPs were identified, yielding 52.3 DRPs per 100 prescriptions (95% CI: 50.7–53.9), and an average of 5.03 DRPs per patient (range: 0–15). The primary metric was DRPs per 100 prescriptions, as this allows comparison with published literature. DRPs per patient and DRPs per 100 patient-days are presented as secondary metrics. Of the 400 enrolled patients, 372 (93%) had at least one DRP and 28 (7%) had DRP-free prescriptions. The detailed patient flow is illustrated in Figure 2.

### PCNE classification of drug-related problems

**FIGURE 3:**
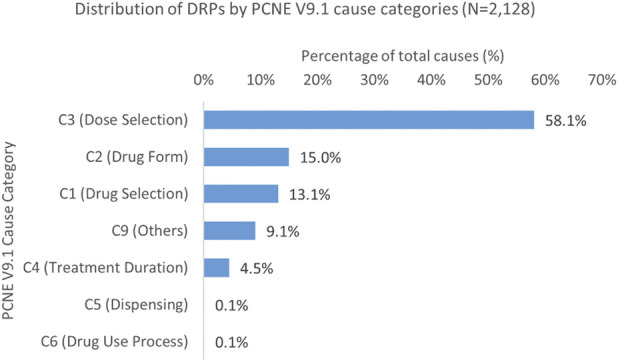
PCNE cause domains of drug-related problems (n = 2,128). Horizontal bar chart showing the distribution of DRPs by PCNE V9.1 cause categories. Dose selection (C3: 58.1%), drug form (C2: 15.0%), drug selection (C1: 13.1%), other/monitoring (C9: 9.1%), treatment duration (C4: 4.5%), dispensing (C5: 0.1%), and drug use process (C6: 0.1%).

Table 2 presents the distribution of DRPs according to PCNE classification. Treatment effectiveness problems (P1) accounted for 86.7% (n = 1,742), with 53.4% showing no effect of treatment (P1.1) and 43.2% due to suboptimal drug effect (P1.2). Treatment safety problems (P2) constituted 12.4% (n = 250). The major cause of DRPs was dose selection errors (C3: 58.13%, n = 1,208), mainly due to under-dosing (C3.1: 40.2%) and over-dosing (C3.2: 43.7%). Inappropriate drug form (C2.1) was observed in 15.01% (n = 312) of prescriptions, followed by 13.08% (n = 278) errors related to drug selection (C1), with 52.3% due to excessive polypharmacy (C1.6). Monitoring errors (C9.1) accounted for 9.1% (n = 169), treatment duration errors (C4) were 0.48% (n = 10), and dispensing errors (C5) and drug use process (C6) errors were minimal (0.09% each). Figure 3 illustrates the distribution of PCNE cause domains.

**TABLE 2:** PCNE classification of DRPs (n = 2,010).

PCNE domain	Code	Description	N	%
Problems (P)				
Treatment effectiveness	P1	​	1,742	86.7
​	P1.1	No effect of drug treatment despite correct use	930	53.4
​	P1.2	Effect of drug treatment not optimal	752	43.2
​	P1.3	Untreated symptoms or indication	60	3.4
Treatment safety	P2	​	250	12.4
​	P2.1	Adverse drug event	122	48.8
​	P3.1	Unnecessary drug treatment	128	51.2
Other	P3	​	18	0.9
​	P3.2	Unclear problem requiring clarification	18	0.9
Causes (C)	​	​	2,128*	​
Drug selection	C1	​	278	13.08
​	C1.1	Inappropriate drug according to guidelines	22	8.09
​	C1.2	No indication for drug	8	2.94
​	C1.3	Inappropriate combination	56	20.59
​	C1.4	Inappropriate duplication	40	14.71
​	C1.5	No/incomplete drug treatment despite indication	4	1.47
​	C1.6	Too many different drugs prescribed	142	52.21
Drug form	C2	​	312	15.01
​	C2.1	Inappropriate drug form/formulation	312	100
Dose selection	C3	​	1,208	58.13
​	C3.1	Drug dose too low	486	40.2
​	C3.2	Drug dose too high	528	43.7
​	C3.3	Dosage regimen not frequent enough	124	10.3
​	C3.4	Dosage regimen too frequent	68	5.6
​	C3.5	Dose timing instructions wrong/unclear	2	0.2
Treatment duration	C4	​	10	0.48
​	C4.1	Duration too short	2	20
​	C4.2	Duration too long	8	80
Dispensing	C5	​	2	0.09
​	C5.1	Prescribed drug not available	2	100
Drug use process	C6	​	2	0.09
​	C6.1	Inappropriate timing of administration	2	100
Other	C9	​	278	13.08
​	C9.1	No/inappropriate outcome monitoring (incl. TDM)	250	92
​	C9.2	Other cause	22	8

* Total causes (2,128) exceed total DRPs (2,010) because multiple causes could apply to a single DRP. DDI, drug-drug interaction; PCNE, Pharmaceutical Care Network Europe.

### Dose-related DRPs: detailed analysis

Dose-related DRPs comprised 58.13% of overall DRPs. Table 3 provides the distribution of dose-related DRPs by the drug class. Antibiotics accounted for 67.3% (n = 813) of all dosing related DRPs, followed by supportive medications (antipyretics/analgesics) at 18.0% (n = 218), and antiemetics/others at 14.2% (n = 177).

**TABLE 3:** Dose related DRPs by drug class (n = 1,208).

Drug class	Underdose n (%)	Overdose n (%)	Wrong frequency n (%)	Total n (%)
Antibiotics	326 (67.1)	355 (67.2)	129 (66.5)	813 (67.3)
Antipyretics/Analgesics	87 (17.9)	95 (18.0)	36 (18.6)	218 (18.0)
Antiemetics	36 (7.4)	39 (7.4)	14 (7.2)	89 (7.4)
Others	37 (7.6)	39 (7.4)	15 (7.7)	88 (7.3)

Percentages are row percentages within each drug class. “Others” include antiemetics, antipyretics/analgesics not specified, and supportive care medications.

Examples of dose-related DRPs included: ceftriaxone prescribed at 50 mg/kg/day instead of 80–100 mg/kg/day for a meningitis patient (under-dose, C3.1); paracetamol prescribed at 20 mg/kg/dose instead of 10–15 mg/kg/dose (overdose, C3.2); vancomycin prescribed without monitoring drug levels (C9.1); amoxicillin prescribed once daily instead of twice daily (C3.3).

### PCNE intervention classification (I)

A total of 1,986 interventions were suggested by the pharmacist for 2,010 DRPs (elaborated in Table 4). Drug-level interventions (I3) were 65.6% (n = 1,302), of which 53.8% were dose adjustments, 26.9% were formulation changes, and 9.4% were related to drug discontinuation. Prescriber-level interventions (I1) constituted 33.3% (n = 662); of these, 80.4% were discussed directly with the prescriber during ward rounds, and in 10.0% of cases interventions were proposed to the prescriber through written note in the medical record. Patient-level interventions (I2) were limited to 0.9% (n = 18), mainly involving referral of patients to the prescriber.

**TABLE 4:** PCNE classification of interventions (n = 1,986).

PCNE domain	Code	Description	N	%
Prescriber level	I1	​	662	33.3
​	I1.1	Prescriber informed only	50	7.6
​	I1.2	Prescriber asked for information	14	2.1
​	I1.3	Intervention proposed to prescriber	66	10.0
​	I1.4	Intervention discussed with prescriber	532	80.4
Patient level	I2	​	18	0.9
​	I2.1	Patient (drug) counseling	2	11.1
​	I2.2	Written information provided	4	22.2
​	I2.3	Patient referred to prescriber	10	55.6
​	I2.4	Spoken to family member/caregiver	2	11.1
Drug level	I3	​	1,302	65.6
​	I3.1	Drug changed	52	4.0
​	I3.2	Dosage changed	701	53.8
​	I3.3	Formulation changed	350	26.9
​	I3.4	Instructions for use changed	72	5.5
​	I3.5	Drug paused or stopped	123	9.4
​	I3.6	Drug started	4	0.3
Other	I4	​	4	0.2
​	I4.1	Other intervention	3	75.0
​	I4.2	Side effect reported to authorities	1	25.0

PCNE, Pharmaceutical Care Network Europe. Total interventions (1,986) are fewer than total DRPs (2,010) because 24 DRPs received no intervention (patient was discharged or error already corrected by prescriber).

Of the 2,010 identified DRPs, 1,986 (98.8%) received pharmacist interventions. The remaining 24 DRPs (1.2%) did not receive interventions because the DRP was already corrected by the prescriber before pharmacist review (n = 16) or the patient had been discharged before an intervention could be made (n = 8).

### PCNE acceptance (A) and resolution (O) status

Acceptance (A) and resolution (O) status according to PCNE is presented in Table 5. Pharmacist interventions demonstrated 98.7% (n = 1,960) acceptance (A1), of which 89.1% were fully implemented and 9.6% were partially implemented. Only 1.3% (n = 26) of the interventions were not accepted (A2), primarily due to non-agreement with the prescriber. The interventions were monitored for their resolution status (O), 81.1% (n = 1,631) of the identified problems were totally resolved (O1.1), and 13.9% were partially resolved (O2.1), while 5.0% remained unresolved (O3), most of them due to lack of patients’ and prescribers’ compliance.

**TABLE 5:** PCNE acceptance and resolution status of interventions.

PCNE domain	Code	Description	N	%
Acceptance (A)	​	​	1,986	​
Accepted	A1	​	1,960	98.7
​	A1.1	Accepted and fully implemented	1,770	89.1
​	A1.2	Accepted and partially implemented	190	9.6
​	A1.3	Accepted but not implemented	8	0.4
​	A1.4	Accepted, implementation unknown	2	0.1
Not accepted	A2	​	26	1.3
​	A2.1	Not accepted: not feasible	8	30.8
​	A2.2	Not accepted: no agreement	14	53.8
​	A2.3	Not accepted: other reason	4	15.4
Resolution status (O)	​	​	1,856	​
Solved	O1	​	1,631	81.1
​	O1.1	Problem totally solved	1,631	81.1
Partially solved	O2	​	258	13.9
​	O2.1	Problem partially solved	258	13.9
Not solved	O3	​	93	5.0
​	O3.1	Lack of patient cooperation	36	38.7
​	O3.2	Lack of prescriber cooperation	26	28.0
​	O3.3	Intervention not effective	16	17.2
​	O3.4	No need/possibility to solve	15	16.1

PCNE: Pharmaceutical Care Network Europe. Acceptance categories: A1 = accepted, A2 = not accepted. Resolution categories: O1 = solved, O2 = partially solved, O3 = not solved. Percentages are column percentages.

### Severity of drug-related problems

**FIGURE 4:**
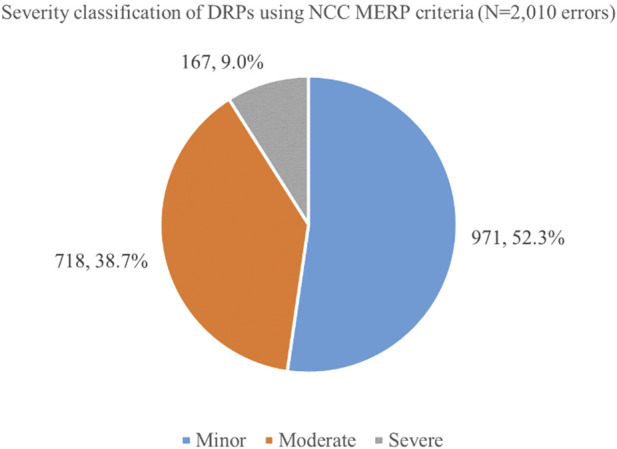
Severity of drug-related problems (NCC MERP classification). Bar chart showing severity distribution of 2,010 DRPs: minor (52.3%, n = 1,094), moderate (38.7%, n = 777), and severe (9.0%, n=184).

Severity assessment of the errors using NCC MERP criteria (Figure 4) classified the 2,010 errors as 52.3% (n = 1,094) minor, 38.7% (n = 777) moderate, and 9.0% (n = 184) severe. Severe errors included prescription of vancomycin without therapeutic drug monitoring (nephrotoxicity risk), co-administration of IV ceftriaxone and calcium (precipitation risk), aminoglycoside high doses without renal monitoring, contraindicated drug combinations and tenfold dosing errors in low-weight infants.

### Cost impact of pharmacist interventions

Pharmacist-led interventions were associated with total cost savings of PKR 363,184.31 (approximately USD 1,290) over 10 months, with a 23.1:1 positive to negative cost ratio, demonstrating that for every PKR 1 spent on interventions that increased costs (therapeutically necessary changes), PKR 23 were saved through DRP corrections. Cost impact analysis was performed on 1,423 interventions that had direct cost implications. The remaining 563 interventions (e.g., dose frequency adjustments without cost change, therapeutic drug recommendations, patient counseling) had no direct medication cost impact and were excluded from the cost saving calculation. These interventions are clinically important but their economic benefit is indirect (e.g., preventing adverse events, reducing length of stay) and was not quantified in this analysis. The weighted average cost savings for 1,423 interventions amounted to an average of PKR 255 per intervention. The largest savings came from wrong dose correction (C3): 50.5% (PKR 262/intervention). Duplication of therapy (C1.4) accounted for 20.8% (average PKR 1,888/intervention, highest yield), while drug formulation changes (C2.1) accounted for 15.3% (average PKR 159/intervention). The projected annual savings = PKR 363,184.31 × (12/10) = PKR 435,821.17 (approximately USD 1,556). Per intervention analysis showed duplication of therapy and inappropriate drug choice as the highest economic yield, while dose frequency adjustments showed the lowest yield as shown in Table 6.

**TABLE 6:** Direct cost savings from correction of DRPs.

Intervention category	PCNE cause	N	Positive cost savings (PKR)	Negative costs (PKR)	Net savings (PKR)	% of total savings
Wrong dose correction	C3	701	186,532.12	2,985.00	183,547.12	50.5%
Duplication of therapy	C1.4	40	75,532.11	—	75,532.11	20.8%
Drug formulation change	C2.1	350	59,367.34	3,789.00	55,578.34	15.3%
Inappropriate drug combinations	C1.3	56	14,278.13	—	14,278.13	3.9%
Treatment duration adjustment	C4	84	19,988.80	7,543.22	12,445.58	3.4%
Dose frequency adjustment	C3.3/C3.4	172*	13,362.13	2,102.20	11,259.93	3.1%
Inappropriate drug choice	C1.1	20	10,543.10	—	10,543.10	2.9%
TOTAL	​	1423^†^	379,603.73	16,419.42	363,184.31	100%

* Dose frequency adjustments: C3.3 (n = 112) + C3.4 (n = 60). Cost per DRP prevented: PKR 241. Return on investment: 142%. PKR: Pakistani Rupees; USD: United States Dollars (1 USD ≈ 280 PKR at time of study). Positive cost savings indicate reduction in medication expenses; negative costs indicate therapeutically necessary increases in drug costs. Net savings = positive savings - negative costs.

^†^ Only 1,423 of 1,986 interventions had direct medication cost implications; the remainder (e.g., dose frequency adjustments without cost change, TDM, recommendations) had no direct cost impact.

Cost per DRP prevented was PKR 241 (USD 0.86), calculated as total net savings (PKR 363,184) divided by number of DRPs fully corrected (n = 1,631). While accounting for the pharmacist time (600 h; PKR 150,000), the estimated net savings after deducting the intervention costs was PKR 213,184, yielding a return on investment of 142%.

### Cost-saving interventions

Supplementary Table 2 presents illustrative high-impact pharmacist interventions. A detailed list of all 1,986 interventions reveals that dose corrections resulted in PKR 183,547, the highest saving. Correction of duplicate therapy resulted in PKR 75,532 by eliminating redundant drug combinations and unnecessary costs. Formulation changes primarily IV-to-oral switches saved PKR 55,578 enabling earlier discharge and interventions linked to unnecessary drugs saved PKR 22,005. The average cost savings per patient was PKR 908 (USD 3.24).

**FIGURE 5:**
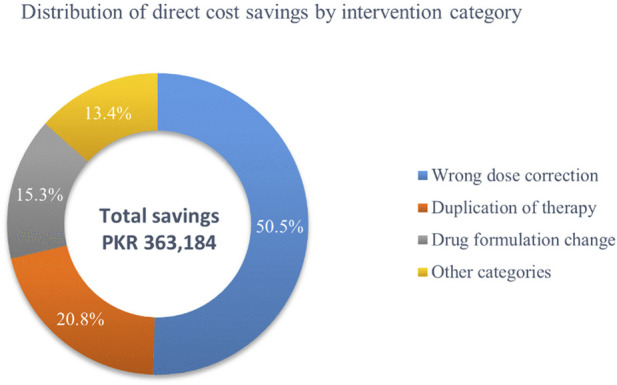
Contribution of intervention categories to total cost savings. Doughnut chart showing proportion of total savings (PKR 363,184): wrong dose correction (50.5%), duplication of therapy (20.8%), drug formulation change (15.3%), and other categories (13.4%).

Figure 5 illustrates the contribution of each category to total cost savings.

Sensitivity analysis confirmed the robustness of the base-case findings (Supplementary Table 3). Total cost savings remained positive across all scenarios, ranging from PKR 326,866 (drug cost −10%) to PKR 399,502 (drug cost +10%). The return on investment ranged from 94% (pharmacist time 2.5 h/day) to 223% (pharmacist time 1.5 h/day). Cost per DRP prevented ranged from PKR 79 to PKR 241 depending on pharmacist time and hourly cost assumptions.

### Predictors of drug-related problems

Univariate logistic regression analysis was performed to identify factors associated with DRPs (Supplementary Table 4). In univariate analysis, length of stay >7 days (OR 3.33, 95% CI: 1.53–7.25, p = 0.003) and urban residence (OR 0.24, 95% CI: 0.09–0.64, p = 0.005) were significantly associated with DRPs. Fever at admission showed borderline significance (OR 3.31, 95% CI: 0.89–12.35, p = 0.075). Past immunization, age, gender and number of prescribed medications showed no statistically significant association in univariate analysis (p > 0.05 for all). In multivariate analysis, four independent predictors of DRPs were identified (Table 7). The model showed good fit (Hosmer-Lemeshow p = 0.55) and acceptable discrimination (AUC = 0.78). Each additional drug increased DRP risk by 32% (AOR 1.32, 95% CI: 1.18–1.48, p < 0.001). Fever at admission was associated with nearly three-fold higher risk (AOR 2.84, 95% CI: 1.12–7.21, p = 0.028). Length of stay >7 days increased risk by 76% (AOR 1.76, 95% CI: 1.21–2.56, p = 0.003). Past immunization remained protective (AOR 0.51, 95% CI: 0.35–0.74, p < 0.001). Age, gender and residence were not significant predictors in the multivariate model (p > 0.05 for all).

**TABLE 7:** Multivariate Analysis of Predictors of Drug-Related problems.

Variable	Adjusted OR	95% CI	p-value
Number of prescribed medications (per additional drug)	1.32	1.18–1.48	<0.001
Fever at admission	2.84	1.12–7.21	0.028
Length of stay >7 days	1.76	1.21–2.56	0.003
Past immunization	0.51	0.35–0.74	<0.001
Age (per year increase)	1.08	0.98–1.19	0.112
Male gender	1.21	0.84–1.74	0.298
Urban residence	1.14	0.79–1.64	0.483

OR, odds ratio; CI, confidence interval; AOR, adjusted odds ratio; LOS, length of stay. Crude ORs, from univariate logistic regression; Adjusted ORs, from multivariate logistic regression adjusting for all variables shown. Hosmer-Lemeshow p = 0.55, AUC, 0.78. Variable selection used backward stepwise elimination with entry criterion p < 0.10 and retention criterion p < 0.05.

**FIGURE 6:**
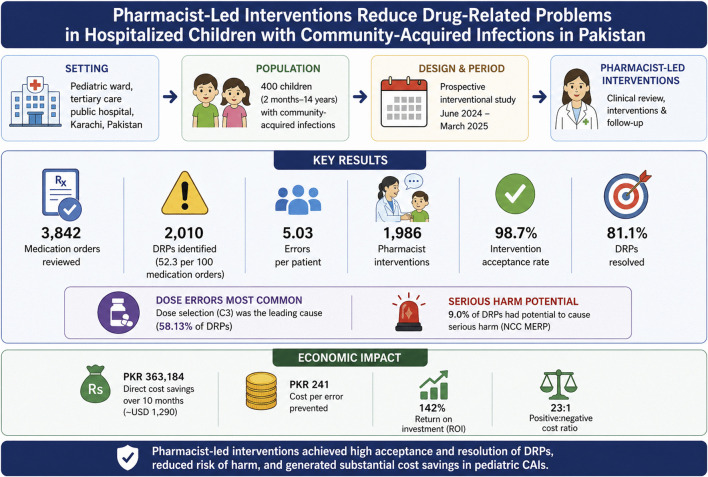
Summary of key findings. Overview of study design, population, key results (DRP prevalence, dose errors, severity, acceptance rate, resolution rate), and economic impact (cost savings, ROI, cost ratio).

The graphical Abstract of the study is presented in Figure 6.

## Discussion

This prospective study of 3,842 medication orders in 400 hospitalized children with CAIs in Pakistan contributes significantly to the literature on pediatric medication safety in LMICs. This study makes several novel contributions to the literature. First, to the best of published literature, this represents the first prospective application of the PCNE V9.1 classification system to identify and categorize DRPs in hospitalized children with CAIs in South Asia. Second, this study is the first from Pakistan to link PCNE-classified DRPs with a comprehensive economic analysis using actual drug acquisition costs rather than estimates, demonstrating positive return on investment for clinical pharmacy services in a low-resource setting. Third, we identify past immunization as a previously unreported protective factor associated with lower odds of DRPs, suggesting that vaccination programs may have an indirect role in medication safety by reducing illness severity and treatment complexity. Fourth, the 98.7% acceptance rate and 81.1% resolution rate of pharmacist interventions provide real-world evidence for successful integration in LMICs. Collectively, these findings suggest that clinical pharmacy services represent a net investment rather than a financial burden [24, 30], providing actionable evidence for hospital administrators and policy makers to improve medication safety [18, 19].

In the current study, 52.3 DRPs were observed per 100 medication orders, and 93% of patients had at least one DRP. This prevalence is higher than reported in some LMIC settings. Feyissa Mechessa et al. (2020) reported 85% in Ethiopian pediatric infectious patients [27], while a systematic review reported pediatric DRP prevalence ranging from 45% to 92% depending on setting and classification criteria [7]. The higher rate observed in the current study may reflect the prospective daily review methodology and the inclusion of all medication orders, including those for supportive care. The mean DRP rate of 5.03 per patient in this study is higher than the 1.3 DRPs per patient reported in a PICU study from Iran [32]. This discrepancy may reflect differences in case mix (CAIs vs. mixed PICU patients), the prospective daily review methodology employed in the current study, the inclusion of all medication orders regardless of perceived severity, and differences in prescribing complexity between settings [33, 34].

In this study, dose selection-related DRPs (C3) accounted for 58.13% (Table 2) which aligns with recent studies, where Shirzad-Yazdi et al. in pediatric ICU in Iran reported 56.8% dose selection errors [32]. Feyissa Mechessa et al. in Ethiopia reported 53.9% dosing errors [27], Ali et al. in a study from Pakistan reported dosing errors as leading DRP category in hospitalized patients [14], and Mi et al. in a global systematic review identified dosing errors as the most frequent pediatric DRP [7]. This difference may reflect local prescribing practices, availability of pediatric formulations, presence of antimicrobial stewardship programs, and the specific patient population studied. In our setting, the high proportion of dose-related DRPs likely reflects the complexity of weight-based dosing in children and the absence of computerized decision support systems [35]. In contrast to this study, a multicenter PICU study from Northwest Ethiopia reported that drug selection DRPs (46.0%) were more frequent than dose selection-related DRPs (43.8%) [36]. This discrepancy may reflect differences in clinical settings and patient populations. The current study included children with CAIs in general pediatric wards, where weight-based dosing for antibiotics is complex and polypharmacy is frequently reported. In contrast, the Ethiopian study was conducted in PICUs, where critically ill children may require more frequent drug selection decisions (e.g., choosing empiric therapy, selecting vasoactive agents, managing multiple organ dysfunction). Other contributing factors may include local prescribing practices, availability of pediatric formulations, presence of antimicrobial stewardship programs, and the absence of computerized decision support systems in our setting [35].

Furthermore the findings of under-dosing (C3.1: 40.2%) and over-dosing (C3.2: 43.7%) related DRPs provide a useful insight (Table 2). Several studies have reported similar rates of dose-related DRPs for prescription of antibiotics in Pakistan [37]. Under-dosing risks treatment failure, prolonged illness and AMR; especially in serious infections like meningitis, under-dosing of beta lactams can lead to sub-therapeutic drug levels and neurological disabilities [38]. Overdosing risks toxicity, adverse effects, and increased monitoring; for example, aminoglycoside over dose without therapeutic drug monitoring can cause permanent hearing loss and nephrotoxicity [39].

DRPs classified as drug-form (C2: 15.01%) can be associated to the lack of pediatric-friendly formulations. As observed in global literature on drug development gaps, this highlights the needs for pharmaceutical industries and hospitals to prioritize age- and weight-appropriate formulations in their formularies [40]. Drug selection DRPs (C1: 13.08%) were mainly due to overprescribing of drugs (C1.6: 52.3%), showing the high rate of polypharmacy (73.5% receiving ≥5 medications). The addition of every single drug increased DRP risk by 32%, which is consistent with the findings of other studies showing polypharmacy as strongest predictor of DRPs [41, 42]. The relationship between polypharmacy and DRPs in this pediatric population can be explained by several factors: (1) increased likelihood of drug-drug interactions requiring complex dose adjustments; (2) higher probability of cumulative dosing related DRPs when calculating multiple weight-based doses; (3) greater monitoring burden leading to missed laboratory assessments; and (4) additive adverse effects that may be misattributed to underlying infection rather than medication toxicity [4, 5, 42, 43]. Monitoring-related DRPs (C9.1: 9.1%) especially failure of therapeutic drug monitoring of vancomycin and aminoglycosides represent missed opportunities of prevention of toxicity [39]. Incorrect antibiotic dosing combined with monitoring related problems has dual clinical implications beyond individual patient harm. Under-dosing may lead to treatment failure, prolonged illness, and antimicrobial resistance, while over-dosing increases risk of toxicity and adverse effects. In the current study, antibiotics constituted 67.3% of all dosing related DRPs, highlighting the need for AMS programs that include systemic pharmacist-led dose verification of antimicrobial prescriptions [37, 44–46].

Table 8 compares the PCNE findings of this study with other recent pediatric studies. The higher proportion of drug form-related DRPs (15.01% vs. 6.2–10.1%) can potentially reflect greater challenges in access to pediatric friendly formulations or strict identification of dosage form errors in the prospective review by the pharmacist [14, 27, 32, 47].

**TABLE 8:** Comparison of PCNE findings with other studies.

Study	Setting	Inclusion criteria	Dose selection (C3)	Drug form (C2)	Drug selection (C1)	Monitoring (C9)
Present study (2026)	Pakistan, pediatric ward	Children 2 months-14 years with CAIs	58.13%	15.01%	13.08%	9.1%
Shirzad-Yazdi et al. [32]	Iran, PICU	Pediatric ICU patients	56.8%	8.2%	18.5%	12.3%
Ali et al. [14]	Pakistan, mixed	Children with bacterial meningitis	52.3%	10.1%	15.2%	14.5%
Feyissa Mechessa et al. [27]	Ethiopia, infectious diseases	Pediatric patients with infectious diseases	53.9%	7.8%	16.4%	11.2%
Ni et al. [47]	China, chronic diseases	Children with chronic disease in primary care	48.5%	6.2%	22.1%	13.8%

CAIs, Community-acquired infections. All comparator studies used PCNE, classification (versions V8.0 to V9.1). Direct comparisons should be interpreted with consideration of differences in clinical settings and patient populations.

This study observed that 9.0% of identified DRPs were severe, with the potential to cause serious harm to patients, aligning with the findings of a systematic review by Hannibal et al., which showed that preventable harm from DRPs remains a critical global challenge [6]. These 184 severe DRPs, had they not been intercepted, could have resulted in permanent harm or death. These DRPs included vancomycin without TDM, co-administration of drugs that precipitate, tenfold higher doses, and contraindicated combinations.

A systematic review conducted in 2020 of pediatric AMS programs by Donà et al. found that only 14.1% of the studies evaluated cost impact [17]. This study directly addresses the gap by providing cost saving data from a LMIC setting. The observed annual cost savings significantly exceed the average salary of a pharmacist in Pakistan, suggesting that these services can be self-funding while simultaneously improving patient outcomes [24]. The exceptionally high 23:1 positive-to-negative cost ratio demonstrates that for every rupee invested in necessary therapeutic changes, twenty-three rupees are saved by correcting prescribing errors. Crucially, as these calculations exclude indirect benefits like reduced LOS or avoided ADRs, they likely represent a conservative estimate of the true economic impact [48, 49].

For example, each IV-to-oral switch (n = 350) not only saved PKR 159 in direct savings for the patient but was also associated with reduced nursing drug administration time, a potential reduction in hospital stay by 1–2 days, and fewer IV line-associated complications, which would multiply the economic impact. Similarly prevention of one case of vancomycin-induced nephrotoxicity via TDM monitoring could save thousands of dollars reducing due to extended hospital stay and dialysis. Thus, the findings likely underestimate the cost impact of the clinical pharmacy services in this study. Similar cost effectiveness has been reported in Spanish PROA-NEN program, where a 27.3% reduction in antimicrobial expenses over 5 years was reported, and the outpatient antimicrobial treatment ASP program in Spain reported savings of €1,069,963 by avoiding hospital admissions [48, 49]. A study from Pakistan in 2021 by Ahmed et al. reported the cost savings due to clinical pharmacy services but did not quanty them in details [24].

Dosing related-DRPs constituted 58.13% of all DRPs and 50.5% of total cost savings (PKR 183,547), reflecting that they were the most frequent type of DRP and that many of the corrected DRPs involved high-cost antibiotics such as vancomycin, meropenem, and ceftriaxone. These errors affect the entire duration of the treatment, and are highly accepted by prescribers in case of intervention. As these dose related-DRPs included both under-dosing and over-dosing, this highlights that not all interventions for dosing related DRPs reduce expenditure; some increase costs but improve the therapeutic effect. Our net savings calculation accounts for both positive and negative cost impact of dosing related DRPs, providing a transparent cost analysis.

The risk factors identified in this study offer opportunities for targeted prevention. Each additional medication prescribed increases the risk of DRPs (32% higher odds per drug) and presents a cost-saving opportunity, highlighting that patients on ≥ 5 medications should be prioritized for daily pharmacist review [42]. Consequently, protocols to reduce polypharmacy and regular medication reconciliation can mitigate the risk of DRPs. The finding that fever at admission was associated with higher odds of DRPs (nearly three-fold odds) may reflect that febrile patients receive more complex antibiotic regimens, increasing DRP opportunities. Implementing strategies such as verifying dose, indication, and duration of therapy, as well as clinical decision support systems, could reduce these DRPs [50]. Longer LOS results in more DRPs (76% higher odds) but also provides more opportunities for pharmacist interventions. To prevent this, clear handoff communication during shift changes and medication reconciliation during patient admission, transfer, and discharge can reduce DRPs [51]. In this study, protective effect of past immunization (49% lower odds) may be indirect: immunized children may have less severe illness or different infection profiles requiring less complex treatment, rather than immunization directly preventing DRPs. This highlights the rationale for strengthening and promoting vaccination programs and improving access for children to reduce treatment complexity and associated DRPs indirectly [52]. Residual confounding by socioeconomic status or healthcare access cannot be excluded.

In this study, the high pharmacist intervention acceptance rate (98.7%) and DRP resolution rate (81.1%) suggest that pharmacists can effectively identify and facilitate correction of DRPs in this setting. These findings align with the systematic reviews indicating pharmacist participation in multidisciplinary pediatric rounds reduces medication errors by 50%–80% [53, 54]. The high acceptance rate (98.7%) compared with 84% reported in some LMIC studies [27], may be attributed to the daily presence of the pharmacist during ward rounds, which facilitated real-time communication and trust-building with prescribers. Additionally, supervision by an ID specialist and senior hospital pharmacist for complex antimicrobial cases may have enhanced credibility. However, observer bias and the Hawthorne effect (where prescribers may have altered their behavior due to awareness of being observed) cannot be excluded as contributing factors, which could have led to higher acceptance rates than would occur under routine clinical practice, potentially overestimating the true acceptability of pharmacist interventions in non-study settings.

The findings of this study provide evidence-based recommendations for different stakeholders: Hospital administrators in Pakistan to invest more in pharmacy services and human resources linked to it, as pharmacists working in wards generate costs savings that exceed salary costs, and 23:1 cost ratio supports the business case of expending clinical pharmacy coverage; Health policy makers should mandate all the hospitals to establish clinical pharmacy services in tertiary care hospitals, including DRP data in hospital quality indicators, adopt PCNE classification as standard tool for medication safety monitoring and allocate resource for pediatric pharmacy services in LMICs; Clinicians to foster the collaboration with pharmacist, prioritize regular medication review of high risk patients (polypharmacy, febrile, prolonged LOS), document indication of medications to facilitate DRP detection and use weight-based dosing references and calculators; Pharmacy educators to strengthen pediatric pharmacotherapy training in the curricula, train pharmacist in PCNE classification and emphasize on cost effectiveness along with clinical outcome of the pharmacist interventions; Researcher to adopt PCNE in medication safety studies to enable meta-analysis, include economic evaluations along with clinical outcomes and report both positive and negative costs for transparency.

This study included a large sample and a comprehensive daily review of 3,842 medication orders. It used validated PCNE V9.1 classification, which allowed for international comparison. The prospective design of the study captured real-time data, and the use of NCC MERP severity classification provided a standard tool. The economic analysis, utilizing actual cost data and transparent accounting of both positive and negative costs savings, provides a comprehensive overview.

Despite several strengths, this study had several limitations. First, the absence of a control group limits the ability to establish a causal relationship between pharmacist-led interventions and observed outcomes; therefore, the reported associations should not be interpreted as causal effects. Second, the single-center design may restrict generalizability of findings to other healthcare settings. Third, the study population was restricted to children with CAIs, which may limit applicability to other pediatric conditions; additionally high-risk patients (ICU, NICU) were excluded.

Fourth, several methodological limitations should be considered. The primary investigator was not blinded to study objectives, which may have introduced observer or misclassification bias, potentially contributing to overestimation of DRP prevalence. Although a 10% random sample was independently verified (PCNE: κ = 0.85, NCC MERP: κ = 0.82), and complex cases were discussed with an ID specialist and a senior hospital pharmacist, bias cannot be completely ruled out. Although ward rounds were already part of routine clinical practice before the study, the Hawthorne effect cannot be completely excluded, as prescribers may have been aware that data were being collected for the study, potentially influencing their behavior. Fifth, the economic analysis captured only direct cost savings and pharmacist time costs, providing a conservative estimate of true economic value. Indirect costs (e.g., reduced length of stay, prevention of adverse events, therapeutic monitoring) and long-term savings (e.g., antimicrobial resistance prevention) were not quantified. No formal cost-effectiveness analysis using QALYs or DALYs was performed, and purchasing power parity adjustments were not applied to currency conversion. Sixth, regression analysis had several constraints: disease severity was not included as a covariate due to the absence of a standardized scoring system (e.g., Pediatric Early Warning Score, PEWS) in the study ward, potentially leading to residual confounding. Age-stratified analysis to assess pharmacokinetic differences across pediatric subgroups was precluded by the low number of patients without DRPs in older children, leading to model instability. Finally, the estimate of pharmacist time (2 h/day) was derived from activity logs but may not capture all indirect activities.

Future multicenter research and cluster randomized controlled trials comparing pharmacist-led versus CPOE only versus education only as DRP reduction strategies would inform clinical importance and resource allocations. While long-term studies linking pharmacist intervention to clinical outcomes (mortality, readmission, LOS) and full economic analysis would strengthen the evidence base.

## Conclusion

DRPs affect 52.3% of medication orders in hospitalized children with CAIs in Pakistan, with DRPs related to dose selection predominating (58%). One in eleven DRPs (9.0%) had serious harm potential, representing 184 potentially life-threatening DRPs intercepted by pharmacists. Pharmacist-led interventions were associated with 98.7% prescriber acceptance and 81.1% resolution, generating direct cost savings of PKR 363,184 (USD 1,290) over 10 months with a 142% return on investment. Number of medications, fever at admission, and prolonged hospital stay were independent predictors of DRPs, while complete immunization was protective. These findings support integrating clinical pharmacy services into pediatric care in Pakistan and other LMICs as a patient-safety and cost-effective strategy, though formal pharmacoeconomic analysis is warranted.

## Data Availability

The original contributions presented in the study are included in the article/Supplementary Material, further inquiries can be directed to the corresponding authors.

## Supplementary material

SUPPLEMENTARY FILE 1 Patient data collection form. Structured form used to collect demographic, clinical, laboratory, medication, and hospitalization data from electronic medical records and patient files.

SUPPLEMENTARY FILE 2 Cost categories analysis formula and references. Detailed formulas for cost savings calculations across different intervention categories, including the cost calculation formula and data sources from DRAP 2023 price list.

SUPPLEMENTARY TABLE 1 Pharmaceutical Care Network Europe (PCNE) classification of drug-related problems V9.1. Summary of the PCNE classification domains including Problems (P), Causes (C), Interventions (I), Acceptance (A), and Status (O).

SUPPLEMENTARY TABLE 2 Comprehensive details of all pharmacist interventions. Detailed breakdown of 1,986 pharmacist interventions by category, including PCNE codes, frequencies, cost impacts, and clinical impacts.

SUPPLEMENTARY TABLE 3 Sensitivity analysis of economic outcomes. One-way sensitivity analysis results varying pharmacist hourly cost, pharmacist time, and drug acquisition costs, showing total cost savings, ROI, and cost per error prevented.

SUPPLEMENTARY TABLE 4 Univariate analysis of predictors of drug-related problems. Crude odds ratios (OR) with 95% confidence intervals and p-values for each predictor variable from univariate logistic regression analysis.

## Glossary

AMR: Antimicrobial Resistance
AMS: Antimicrobial Stewardship
AOR: Adjusted Odds Ratio
AUC: Area Under the Curve
BNFc: British National Formulary for Children
CAIs: Community-Acquired Infections
CI: Confidence Interval
CPOE: Computerized Prescriber Order Entry
DRAP: Drug Regulatory Authority of Pakistan
DRPs: Drug-Related Problems
EMR: Electronic Medical Record
EPV: Events per variable
HDU: High-Dependency Unit
IBM-SPSS: IBM Statistical Package for Social Sciences
ICU: Intensive Care Unit
ID: Infectious Diseases
IQR: Interquartile Range
IRB: Institutional Review Board
IV: Intravenous
LMICs: Low- and Middle-Income Countries
LOS: Length of Stay
NCC MERP: National Coordinating Council for Medication Error Reporting and Prevention
NICU: Neonatal Intensive Care Unit
PCNE: Pharmaceutical Care Network Europe
PHDU: Pediatric High-Dependency Unit
PICU: Pediatric Intensive Care Unit
PKR: Pakistani Rupee
PMW: Pediatric Medical Ward
QALY: Quality-Adjusted Life Year
ROC: Receiver Operating Characteristic
ROI: Return on Investment
SD: Standard Deviation
SPSS: Statistical Package for Social Sciences
TDM: Therapeutic Drug Monitoring
TLC: Total Leukocyte Count
USD: United States Dollar
WHO: World Health Organization
