# Supplementary material for: Drug-related problems and their predictors in pediatric community-acquired infections: the role of pharmacist-led interventions in Pakistan
Source: J Pharm Pharm Sci. 2026 Jul 16;29:16612. doi: 10.3389/jpps.2026.16612 (PMC13422215; doi:10.3389/jpps.2026.16612)
Supplement: Supplementary file 1 [file Supplementaryfile1.docx]

**Patient Data Collection Form**

| Code #: | | Admission date: |  | | | |  |  |  |
| --- | --- | --- | --- | --- | --- | --- | --- | --- | --- |
| Age: | | Gender: Male Female | | | | | | |  |
| Infant | 2-12 month |  | | | | |  |  |  |
| Toddler | 1-3 year |  |  |  |  |  |  |  |  |
| Preschool | 3-5 Year |  |  |  |  |  |  |  |  |
| School age child | 5-12 Year |  | | | | |  |  |  |
| Weight: | | Height: |  | | | |  |  |  |
| Admission Specialty: | | Principal Diagnosis: (Type of CAI Suspected) | | |  |  |  |  |  |
|  |  |  |  |  |  | ICD Code |  |  |  |
| Any Co morbidities: | | Length of Hospital Stay (LOS): | | 01-10  days | 11-20 days | 21-30 days |  |  |  |
| Hypersensitivity / Allergies (Drug/Food/Any other chemical): | | | | Resident: Urban / Rural | | |  |  |  |
| Complaint at time of Admission: | | | | | | | | |  |
| Medication Reconciliation: | | | | | | | | |  |

| CURRENT MEDICATIONS: List down the medication right from time of admission | | | | | | |
| --- | --- | --- | --- | --- | --- | --- |
| S.no | Medication (Generic) | Brand  Name | Dose | Route | Frequency | Start date |
|  |  |  |  |  |  |  |
|  |  |  |  |  |  |  |
|  |  |  |  |  |  |  |
|  |  |  |  |  |  |  |

| **LABORATORY FINDINGS:**  **(Hematology Biochemistry, Liver Function Test, Lipid Profile, Sugar, Kidney Functions Test, Others)** | | | | | | | | | |
| --- | --- | --- | --- | --- | --- | --- | --- | --- | --- |
| Test | Pre Admission (If any) | On the day of Admission | Days  (Use separate sheet if Patient stay for more than 05 days for further LAB finding documentation) | | | | | | |
|  |  |  |  | | | | | | |
|  |  |  | 02 | | 03 | | 04 | | 05 |
|  |  |  |  |  | |  | |  | |
|  |  |  |  |  | |  | |  | |
|  |  |  |  |  | |  | |  | |
|  |  |  |  |  | |  | |  | |
|  |  |  |  |  | |  | |  | |

| **MICROBIOLOGY Cultures** | | | | | |
| --- | --- | --- | --- | --- | --- |
| S.no | Sample / Site | Date of time of sample taken | Micro Organism | Sensitive Antibiotics | Resistant Antibiotics |
|  |  |  |  |  |  |
|  |  |  |  |  |  |
|  |  |  |  |  |  |
|  |  |  |  |  |  |
