# Supplementary material for: Drug-related problems and their predictors in pediatric community-acquired infections: the role of pharmacist-led interventions in Pakistan
Source: J Pharm Pharm Sci. 2026 Jul 16;29:16612. doi: 10.3389/jpps.2026.16612 (PMC13422215; doi:10.3389/jpps.2026.16612)
Supplement: Supplementary file 2 [file Table1.docx]

**Pharmaceutical Care Network Europe (PCNE) Classification of prescription errors V9.1.**

| Domain | Code | Description |
| --- | --- | --- |
| **Problems (P)** | P1 | Treatment effectiveness (no effect, suboptimal effect, untreated indication) |
|  | P2 | Treatment safety (adverse drug event) |
|  | P3 | Other (unnecessary drug, unclear problem) |
| **Causes (C)** | C1 | Drug selection (inappropriate drug, no indication, inappropriate combination, duplication, too many drugs) |
|  | C2 | Drug form (inappropriate formulation) |
|  | C3 | Dose selection (dose too low, dose too high, frequency inadequate, timing error) |
|  | C4 | Treatment duration (too short, too long) |
|  | C5 | Dispensing (drug not available) |
|  | C6 | Drug use process (administration timing error) |
|  | C7 | Patient-related |
|  | C8 | Patient transfer |
|  | C9 | Other (monitoring issues, TDM not ordered) |
| **Interventions (I)** | I0 | No intervention |
|  | I1 | Prescriber level (informed, asked, proposed, discussed) |
|  | I2 | Patient level (counseling, information, referred) |
|  | I3 | Drug level (drug changed, dose changed, formulation changed, instructions changed, drug paused/stopped, drug started) |
|  | I4 | Other |
| **Acceptance (A)** | A1 | Intervention accepted (fully/partially implemented, not implemented, implementation unknown) |
|  | A2 | Intervention not accepted (not feasible, no agreement, other) |
|  | A3 | Unknown |
| **Status (O)** | O0 | Problem status unknown |
|  | O1 | Problem solved |
|  | O2 | Problem partially solved |
|  | O3 | Problem not solved (lack of cooperation, intervention not effective, no need) |
