# Supplementary material for: Drug-related problems and their predictors in pediatric community-acquired infections: the role of pharmacist-led interventions in Pakistan
Source: J Pharm Pharm Sci. 2026 Jul 16;29:16612. doi: 10.3389/jpps.2026.16612 (PMC13422215; doi:10.3389/jpps.2026.16612)
Supplement: Supplementary file 3 [file Table2.docx]

**Comprehensive Details of All Pharmacist Interventions**

| **Intervention Category** | **PCNE Code** | **Specific Intervention** | **N** | **Associated Drug Class** | **Common Indication** | **Acceptance Rate (%)** | **Cost Impact (PKR)** | **Clinical Impact** |
| --- | --- | --- | --- | --- | --- | --- | --- | --- |
| **Prescriber-Level (I1)** |  |  | **662** |  |  |  |  |  |
|  | I1.1 | Prescriber informed only | 50 | Various | Information sharing | 100% | N/A | Awareness |
|  | I1.2 | Prescriber asked for information | 14 | Various | Clarification | 100% | N/A | Enhanced communication |
|  | I1.3 | Intervention proposed to prescriber | 66 | Various | Dose/ drug change | 95% | N/A | Error prevention |
|  | I1.4 | Intervention discussed with prescriber | 532 | Various | Real-time correction | 99% | N/A | Collaborative care |
| **Patient-Level (I2)** |  |  | **18** |  |  |  |  |  |
|  | I2.1 | Patient (drug) counseling | 2 | Antibiotics | Adherence | 100% | N/A | Improved compliance |
|  | I2.2 | Written information provided | 4 | Multiple | Education | 100% | N/A | Enhanced understanding |
|  | I2.3 | Patient referred to prescriber | 10 | Various | Further discussion | 90% | N/A | Appropriate follow-up |
|  | I2.4 | Spoken to family member/caregiver | 2 | Multiple | Caregiver education | 100% | N/A | Family engagement |
| **Drug-Level (I3)** |  |  | **1,302** |  |  |  |  |  |
|  | I3.1 | Drug changed | 52 | Antibiotics (70%) | Inappropriate choice | 96% | 10,543 | Optimized therapy |
|  | I3.2 | Dosage changed | 701 | Antibiotics (67%) | Under/overdose | 98% | 183,547 | Accurate dosing |
|  | I3.3 | Formulation changed | 350 | Antibiotics (60%) | IV to oral switch | 97% | 55,578 | Earlier discharge |
|  | I3.4 | Instructions changed | 72 | Multiple | Timing/ administration | 94% | N/A | Correct usage |
|  | I3.5 | Drug paused or stopped | 167 | Antibiotics (80%) | Duplication/ unnecessary | 96% | 75,532 | Reduced polypharmacy |
|  | I3.6 | Drug started | 4 | Antibiotics | Omitted essential drug | 100% | (2,985)* | Complete therapy |
| **Other (I4)** |  |  | **4** |  |  |  |  |  |
|  | I4.1 | Other intervention | 3 | Various | Miscellaneous | 100% | N/A | Varied |
|  | I4.2 | Side effect reported | 1 | Various | ADR monitoring | 100% | N/A | Safety reporting |
| *Negative costs (in parentheses) represent therapeutically necessary increases in medication expenses  **Abbreviations:** ADR = Adverse Drug Reaction; TDM = Therapeutic Drug Monitoring; IV = Intravenous; N/A = Not applicable (no direct medication cost change) | | | | | | | | |
