# Supplementary material for: Drug-related problems and their predictors in pediatric community-acquired infections: the role of pharmacist-led interventions in Pakistan
Source: J Pharm Pharm Sci. 2026 Jul 16;29:16612. doi: 10.3389/jpps.2026.16612 (PMC13422215; doi:10.3389/jpps.2026.16612)
Supplement: Supplementary file 4 [file Table3.docx]

**Sensitivity Analysis of Economic Outcomes**

| Scenario | Total Cost Savings (PKR) | Pharmacist Cost (PKR) | Net Savings (PKR) | ROI (%) | Cost per Error Prevented (PKR) |
| --- | --- | --- | --- | --- | --- |
| Base case | 363,184 | 150,000 | 213,184 | 142 | 241 |
| Pharmacist cost PKR 200/hr | 363,184 | 120,000 | 243,184 | 203 | 84 |
| Pharmacist cost PKR 300/hr | 363,184 | 180,000 | 183,184 | 102 | 126 |
| Pharmacist time 1.5 hr/day | 363,184 | 112,500 | 250,684 | 223 | 79 |
| Pharmacist time 2.5 hr/day | 363,184 | 187,500 | 175,684 | 94 | 132 |
| Drug cost +10% | 399,502 | 150,000 | 249,502 | 166 | 241 |
| Drug cost -10% | 326,866 | 150,000 | 176,866 | 118 | 241 |
| *Base case assumptions: pharmacist hourly cost = PKR 250, pharmacist time = 2 hours/day, 300 working days, drug acquisition costs from DRAP 2023 price list. ROI = Return on Investment = (Net Savings ÷ Pharmacist Cost) × 100. Cost per error prevented = Pharmacist Cost ÷ 1,423 errors corrected. All values are reported in Pakistani Rupees (PKR); 1 USD ≈ 280 PKR at the time of study.  †One-way sensitivity analyses varied each parameter independently while holding all other parameters at base case values. | | | | | |
