# Supplementary material for: Drug-related problems and their predictors in pediatric community-acquired infections: the role of pharmacist-led interventions in Pakistan
Source: J Pharm Pharm Sci. 2026 Jul 16;29:16612. doi: 10.3389/jpps.2026.16612 (PMC13422215; doi:10.3389/jpps.2026.16612)
Supplement: Supplementary file 5 [file Table4.docx]

**Univariate Analysis of Predictors of Drug-Related Problems**

| Variable | Crude OR (95% CI) | p-value |
| --- | --- | --- |
| Number of prescribed medications (per additional drug) | 1.24 (0.57-2.70) | 0.593 |
| Fever at admission | 3.31 (0.89-12.35) | 0.075 |
| Length of stay >7 days | 3.33 (1.53-7.25) | 0.003 |
| Past immunization | 1.66 (0.76-3.60) | 0.203 |
| Age (per year increase) | 1.08 (0.98-1.20) | 0.136 |
| Male gender | 1.01 (0.45-2.25) | 0.980 |
| Urban residence | 0.24 (0.09-0.64) | 0.005 |
| Reference categories: Fever (No), LOS ≤7 days, Past immunization (No), Female gender, Rural residence. | | |
