# Supplementary material for: Drug-related problems and their predictors in pediatric community-acquired infections: the role of pharmacist-led interventions in Pakistan
Source: J Pharm Pharm Sci. 2026 Jul 16;29:16612. doi: 10.3389/jpps.2026.16612 (PMC13422215; doi:10.3389/jpps.2026.16612)
Supplement: Supplementary file 6 [file Supplementaryfile2.docx]

**Cost Categories Analysis formula and references:**

1. **Wrong dose corrections (C3):**

Dose reductions: Savings = (Original dose cost - Reduced dose cost) × duration

Dose increases: Negative cost (added expense) included for completeness

1. **Duplication of therapy (C1.4):**

Savings = Full cost of discontinued redundant drug

1. **Drug formulation changes (C2.1):**

IV to oral switch: Savings = (IV cost - Oral cost) × duration

Formulation optimization: Calculated cost difference

1. **Inappropriate drug choice (C1.1):**

Savings = (Original drug cost - Recommended drug cost) × duration

1. **Inappropriate drug combinations (C1.3):**

Savings from discontinuing unnecessary component

1. **Dose frequency adjustments (C3.3, C3.4):**

Savings from reduced administration frequency where applicable

1. **Treatment duration adjustments (C4):**

Shortened duration: Savings from avoided doses

Extended duration: Negative cost where therapeutically necessary

**Cost Calculation Formula:**

Cost Saving = Recommended Drug Cost – Original Drug Cost

Drug Cost = Drug Acquisition Cost × Daily Dose × Duration of Therapy

**Data Sources for Drug Costs:**

Drug Regulatory Authority of Pakistan (DRAP) 2023 price list

Hospital pharmacy cost records for verification

All costs reported in Pakistani Rupees (PKR)
